# Prospects for the Creation of a Photocontrolled Supramolecular Machine Based on a 1,4-Di(azastyryl)benzene Derivative and Cucurbit[7]uril

**DOI:** 10.3390/molecules31091464

**Published:** 2026-04-28

**Authors:** Levon S. Atabekyan, Vitaly G. Avakyan, Vyacheslav N. Nuriev, Alexei V. Medved’ko, Sergey Z. Vatsadze, Sergey P. Gromov

**Affiliations:** 1 NRC “Kurchatov Institute”, Academician Kurchatov Square 1, 123182 Moscow, Russia; boyev@mal.ru (V.G.A.); nvn@org.chem.msu.ru (V.N.N.); lexeym@gmail.com (A.V.M.); 2 Department of Chemistry, M. V. Lomonosov Moscow State University, Vorob’evy Gory, 119899 Moscow, Russia; zurabych@gmail.com; 3 N. D. Zelinsky Institute of Organic Chemistry, 47 Leninsky Prosp., 119991 Moscow, Russia

**Keywords:** di(azastyryl)benzene, cucurbit[7]uril, dimerization, laser photolysis, *E*-*Z* isomerization, triplet state

## Abstract

The photophysical processes and photochemical reactions of 1,4-di(azastyryl)benzene (**1**) derivative {[(*E*,*E*)-**1**](ClO_4_)_2_} were investigated by absorption, luminescence, and laser kinetic spectroscopy in the water solution. The observed photo processes include dimerization, *E*-*Z* isomerization, and intersystem crossing to the triplet state, as well as the complexation [(*E*,*E*)-**1**](ClO_4_)_2_ with cucurbit[7]uril (CB[7]). The [(*E*,*E*)-**1**](ClO_4_)_2_ dye dimerization was shown to be energetically more favorable in the excited state than in the ground state. The reversible photoinduced migration of the dye dication in the CB[7] cavity takes place as a result of partial exit of the [(*E*,*E*)-**1**]^2+^ from the cavity and its subsequent conversion to the (*E*,*Z)*-isomer in the excited state, which undergoes conversion to the initial complex of {[(*E*,*E*)-**1**]@CB[7]}^2+^ after returning to the ground state. This photoprocess is of interest in relation to the scientific problem of designing photocontrolled supramolecular machines.

## 1. Introduction

Distyrylbenzenes are photoactive compounds that show promise for practical purposes such as being involved in the design of organic light-emitting diodes, acting as materials for solar batteries, serving as non-linear optical materials, and use in chemical sensors [1,2,3,4,5,6]. The two C=C double bonds present in the distyrylbenzene molecule give rise to additional possibilities for phototransformations. In particular, these compounds can undergo [2+2]-photocycloaddition reaction accompanied by a considerable change in the molecular geometry, which makes it possible to design supramolecular machines and microrobots responding to light [7,8]. This approach is of particular interest for practical applications because it provides the possibility of contactless remote control. Distyrylbenzenes are addressed in quite a few publications [9,10,11,12,13,14,15,16,17,18,19,20]; however, there are few works [21,22,23,24,25] devoted to the study of photoprocesses in diazadistyrylbenzenes and their complexes with cucurbiturils. Varying the substituents in the diazadistyrylbenzene and the concentrations of the interacting molecules allows us to change the composition of the host–guest complex, thus changing the fluorescent properties of the complex [22,23].

A simple photocontrolled supramolecular machine was obtained in our study from the inclusion complex of a styryl dye with CB[7] [26]. The considerable increase in the fluorescence lifetime of the dye was attributed to the physical motion of the dye cation deep into the cavity in the first several picoseconds; that is, the system was able to operate in the cyclic mode. A more intricate photocontrolled supramolecular machine was based on the pseudorotaxane complex of CB[8] with diquinolylethylene derivative [27]. In this case, exposure to light results in the complex formation of the unsaturated compound in the *cis*-form (*Z*) with CB[8]. It was found that the CB[8] cavity can accommodate two styryl dye molecules. Therefore, the [2+2]-PCA reaction between them can be induced by irradiation; hence, a light-controlled supramolecular assembler based on CB[8] can be generated [25,28]. A small amount of CB[8] and irradiation are sufficient for complete stereospecific conversion of the dyes to cyclobutanes. The investigation and observation of the physical motions and translocations of components in the pseudorotaxane inclusion complexes of cavitands are intriguing aspects of the design of supramolecular machines [29].

The object of our research is (E,E)-1,4-di(4-pyridylvinyl)benzene derivative {[(E,E)-1](ClO4)2} and its inclusion complexes with cucurbit[7]uril (CB[7]). The purpose of this study was to investigate the photonics of this compound and the features of its complexation with CB[7] using absorption, fluorescence, and laser kinetic spectroscopy. In addition to the experimental measurements, quantum chemical calculations were carried out in order to determine the chromophore structure and the structure of the CB[7] complex in the ground and excited states; interpret the experimental absorption and fluorescence spectra; and study the *E-Z* isomerization reaction, which is of interest in connection with the scientific task of creating photocontrolled molecular machines [30,31,32,33,34,35,36].

## 2. Results and Discussion

**Figure 1:**
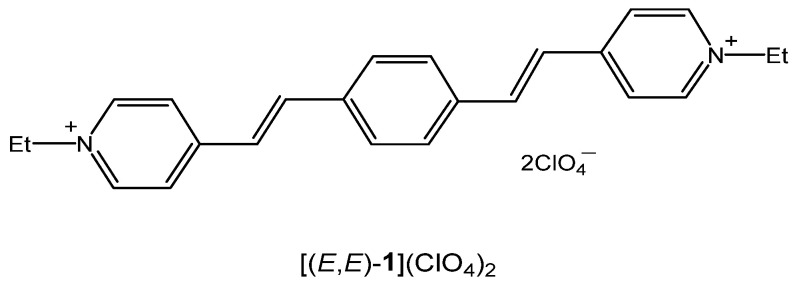
Structure of compound [(*E*,*E*)-**1**](ClO_4_)_2_.

Figure 1 shows the structure of the investigated molecule.

### 2.1. Absorption and Fluorescence Spectra

**Figure 2:**
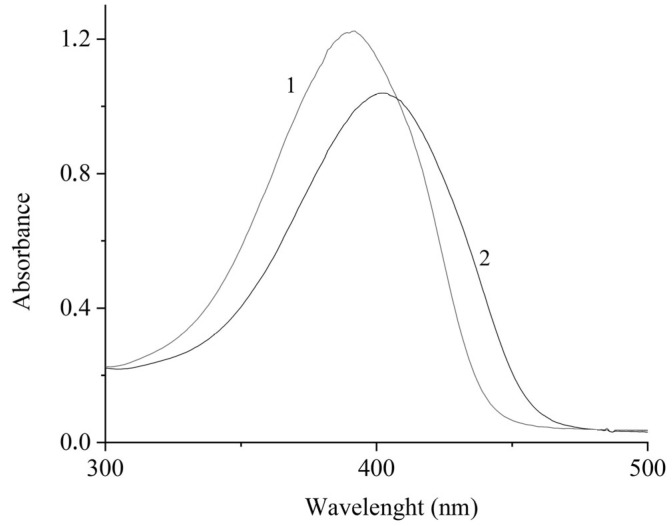
Absorption spectrum of [(*E*,*E*)-1](ClO4)2 in the absence (1) and in the presence (2) of CB[7]. The concentration of [(E,E)-1](ClO4)2 was 2.5 × 10−5; CB[7]—2.5 × 10^−4^ mol/L.

In aqueous solution, dye [(*E*,*E*)-**1**](ClO_4_)_2_ exhibits an absorption band with a maximum at 390 nm. When the host CB[7] molecule is added to a solution of the guest, the long-wavelength absorption band is considerably red-shifted and decreases in intensity (Figure 2).

**Figure 3:**
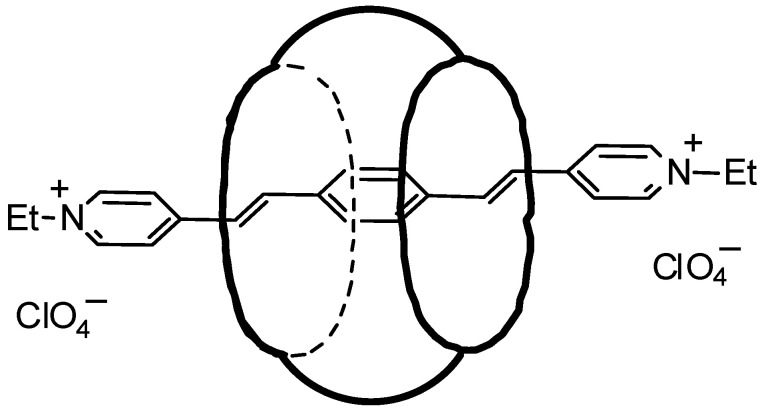
Probable structure of the complex [(*E*,*E*)-**1**]@CB[7](ClO4)_2_.

This attests to the formation of an inclusion complex of CB[7] with the guest, [(*E*,*E*)-**1**](ClO_4_)_2_, characterized by a pseudorotaxane structure in which the cavitand partly shields the guest chromophore from the external medium (Figure 3).

**Figure 4:**
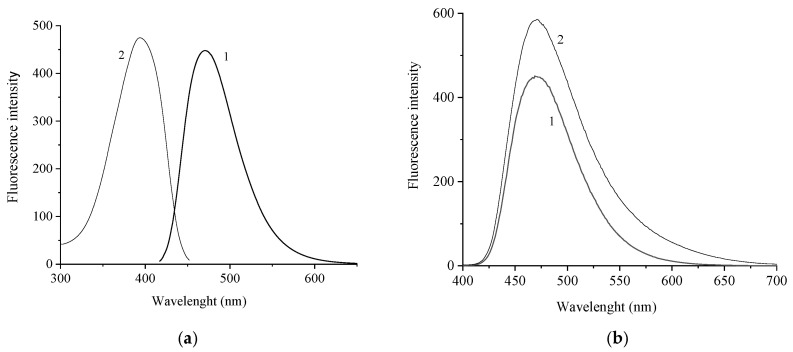
(**a**) Fluorescence (1) and fluorescence excitation (2) spectra of [(*E*,*E*)-**1**](ClO_4_)_2_. (**b**) Fluorescence spectrum of [(*E*,*E*)-**1**](ClO_4_)_2_ in the absence (1) and in the presence (2) of CB[7]. The concentration of [(E,E)-1](ClO4)2 was 2.5 × 10−6; CB[7]—2.5 × 10−^4^ mol/L.

Figure 4 shows the fluorescence spectrum and fluorescence excitation spectrum of [(*E*,*E*)-**1**](ClO_4_)_2_ and fluorescence spectra of [(*E*,*E*)-**1**](ClO_4_)_2_ in the absence and in the presence of CB[7].

The fluorescence excitation spectrum of [(*E*,*E*)-**1**](ClO_4_)_2_ coincides with the absorption spectrum. The Stokes shift of the fluorescence and fluorescence excitation maxima is 75 nm. The wavelength of the major 0-0 electronic transition (λ_00_) = 435 nm. The formation of inclusion complexes is accompanied by an increase in the intensity and broadening of the fluorescence band. The position of the fluorescence band maximum virtually does not change. The change in the fluorescence spectrum upon the formation of the inclusion complex is probably related to the hydrophobic properties of the CB[7] cavity, which shields the guest molecule from the aqueous medium and thus partially blocks non-radiative relaxation channels of the excited state of the dye.

**Figure 5:**
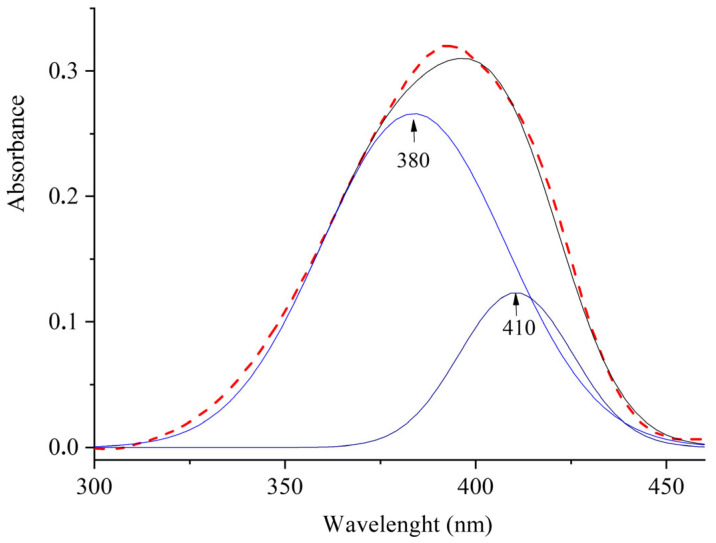
Absorption spectra and Gauss deconvolution for [(*E*,*E*)-**1**](ClO_4_)_2_. The dashed line shows the experimental absorption curve.

Deconvolution of the absorption band of [(*E*,*E*)-**1**](ClO_4_)_2_ (Figure 5) indicates the presence of two bands at λ_max_ = 380 nm and λ_max_ = 410 nm, which can be assigned to absorption of the monomer [(*E*,*E*)-**1**](ClO_4_)_2_ and its dimer, respectively, with the dimer band being red-shifted relative to the monomer band.

**Figure 6:**
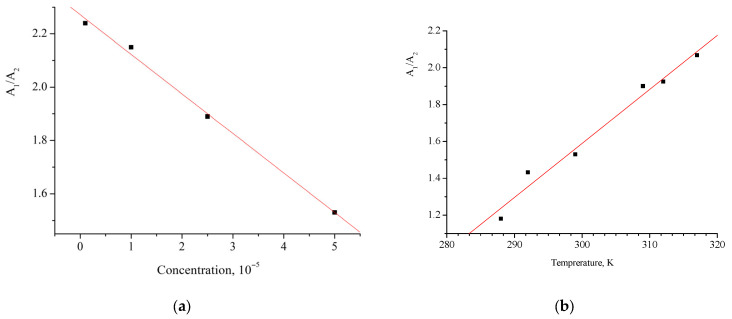
Concentration (**a**) and temperature (**b**) dependences of the integral absorbances at the absorption maxima of [(*E*,*E*)-**1**](ClO_4_)_2_, assigned to the monomer (A_1_) and the dimer (A_2_), respectively.

To make sure that the absorption band with a peak at 410 nm corresponds to the dimer, we studied the concentration and temperature dependences of the integral intensity ratio A_1_/A_2_ for the bands assigned to the [(*E*,*E*)-**1**](ClO_4_)_2_ monomer and dimer, respectively (Figure 6).

It can be seen from Figure 6a that the A_1_/A_2_ value, which characterizes the contribution of absorption of the [(*E*,*E*)-**1**](ClO_4_)_2_ monomer to the total band intensity, increases as the sample concentration decreases. Also, the A_1_/A_2_ ratio increases with the increasing temperature of the solution (Figure 6b). This is precisely the behavior that would be expected under the assumption that the dimerization process is subject to equilibrium:

(1) M+M⇄ D

### 2.2. Laser Kinetic Spectroscopy

**Figure 7:**
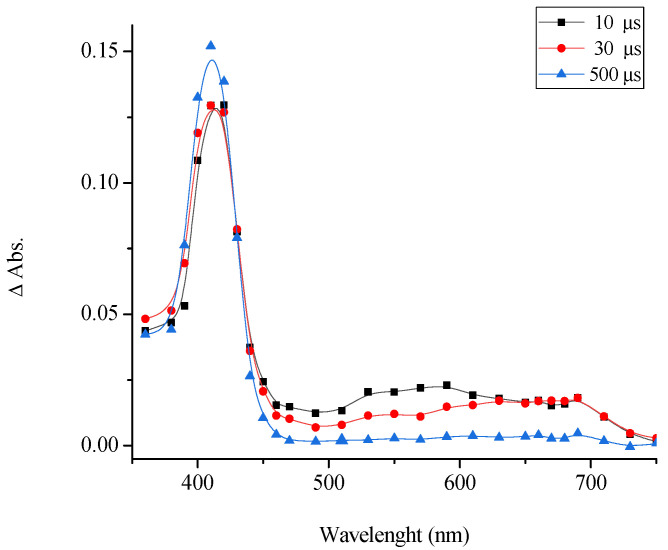
Difference photoinduced absorption spectra of an oxygen-free solution of [(*E*,*E)*-**1**](ClO_4_)_2_ measured 10, 30, and 500 µs after the laser pulse. The concentration of [(*E*,*E*)-**1**](ClO_4_)_2_ was 2.5 × 10^−5^ mol/L.

The pulsed-laser excitation of [(*E*,*E*)-**1**](ClO_4_)_2_ is accompanied by a change in the absorption spectrum shown in Figure 7.

**Figure 8:**
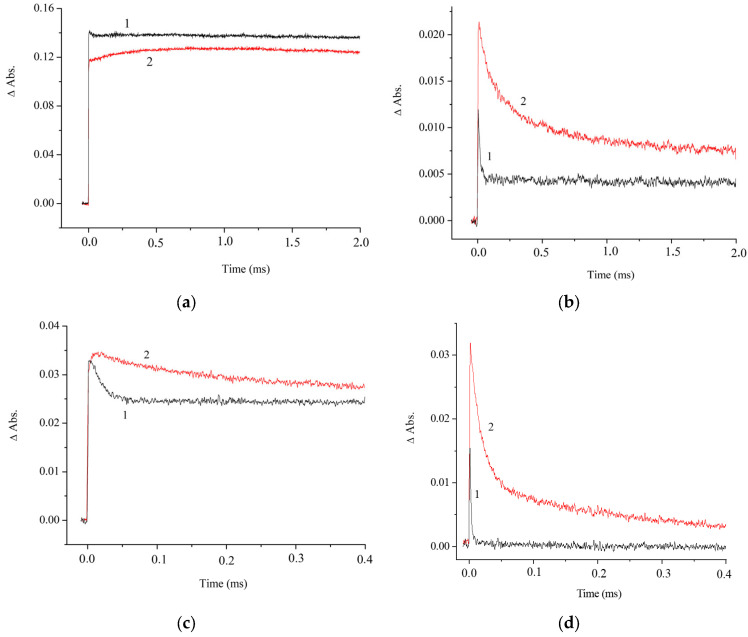
Kinetic curves for variation in the absorbance of air-saturated (1) and air-free (2) solutions of [(*E*,*E*)-**1**](ClO_4_)_2_ at λ = 420 nm (**a**), 450 nm (**b**), 440 nm (**c**), and 570 nm (**d**).

The spectrum of the photoinduced variation in the absorbance shows an intense and relatively narrow absorption band in the 390–460 nm range (λ_max_ = 410 nm) and a broad band in the 500–750 nm range. Figure 8 presents the kinetic curves measured at the maxima of the photoinduced absorption bands

The variation in the absorption in the 350–400 nm range is attributable to the *E*-*Z* photoisomerization. The observed absorption variation in this range may be due to the decrease in the absorbance at the major absorption band of the *E* isomer [(*E*,*E*)-**1**](ClO_4_)_2_ (λ_max_ = 390 nm) and simultaneous increase in the absorbance caused by the formation of the *Z* isomer of the dye [(*E*,*Z*)-**1**](ClO_4_)_2_, which possesses a similar (blue-shifted) absorption spectrum, but with a higher extinction coefficient. Figure 8 shows the kinetic curves for the variation in the photoinduced absorption of air-saturated (1) and air-free (2) solutions of [(*E*,*E*)-**1**](ClO_4_)_2_ for various wavelengths and time scales.

The decrease in the photoinduced absorption at λ = 570 nm (Figure 8d, curve 2) is characterized by a biexponential decay with the rate constants *k*_1_ = 6 × 10^4^ s^−1^ and *k*_2_ = 4 × 10^3^ s^−1^. The effect of air oxygen on the variation kinetics of photoinduced absorption indicates that, apart from the triplet–triplet (T-T) absorption, the formation of a relatively long-lived photoreaction product from the triplet state takes place. The slow stage of the decrease in the induced absorption at λ = 570 nm coincides in time with the relatively slow increase in the absorbance at λ = 420 nm (Figure 8a, curve 2). Meanwhile, the photoproduct with λ_max_ = 420 nm is mainly formed from the singlet excited state (Figure 8a). The lifetime of this product follows a monoexponential dependence with a rate constant of 66 s^−1^.

If we assume that the band at λ_max_ = 410 nm in the photoinduced absorption spectrum belongs to the dye dimer, this means that the dimer concentration increases upon photoexcitation, since the dimer is more stable in the excited state. This assumption is supported by the results of quantum chemical calculations (see below). The biexponential pattern of variation in the absorbance at λ = 570 nm (Figure 8d), which should be assigned to the relaxation of the triplet energy of molecules, can be attributed to the triplet states of both the monomer and dimer of [(*E*,*E*)-**1**](ClO_4_)_2_. The presence of CB[7] does not induce a change in the spectrum or relaxation kinetics of the photoinduced absorption.

### 2.3. Quantum Chemical Calculations

#### 2.3.1. Quantum Chemical Calculation for Monomers of the Dication (**1**)^2+^

**Figure 9:**
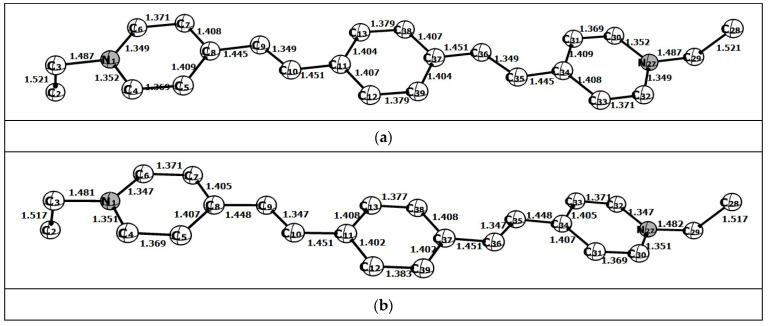

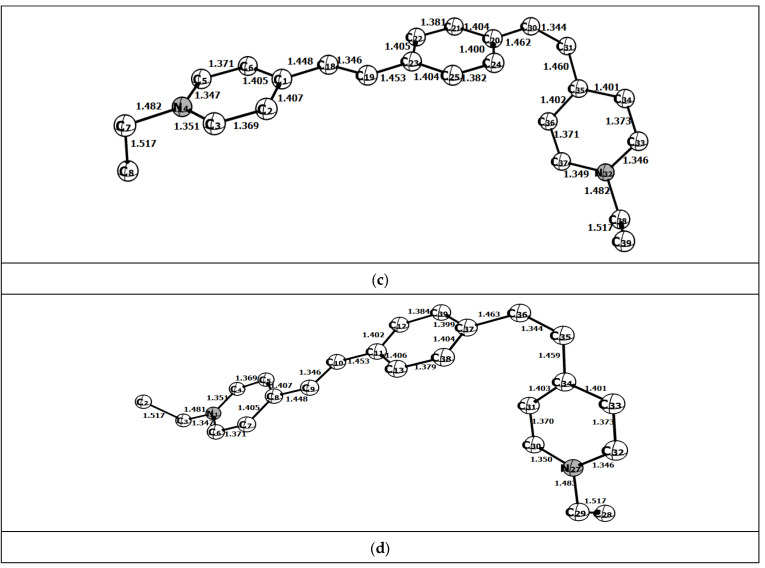
(**a**) Calculated structure of *anti*-[(*E*,*E*)-**1**]^2+^. χC_5_-C_8_-C_9_-C_10_ = −0.1°; χC_8_-C_9_-C_10_-C_11_ = 179.9°; χC_9_-C_10_-C_11_-C_13_ = 0°. (**b**) Calculated structure of *syn*-[(*E*,*E*)-**1**]^2+^. χC_5_-C_8_-C_9_-C_10_ = −1.7°; χC_8_-C_9_-C_10_-C_11_ = 179.7°; χC_9_-C_10_-C_11_-C_13_ = 0°. (**c**) Calculated structure of *anti*-[(*E*,*Z*)-**1**]^2+^. χC_24_-C_20_-C_30_-C_31_ = 35.4°; χC_20_-C_30_-C_31_-C_35_ = 7.7°; χC_30_-C_31_-C_34_-C_36_ = 34.0°. (**d**) Calculated structure of *syn*-[(*E*,*Z*)-**1**]^2+^. χC_38_-C_37_-C_36_-C_35_ = 35.3°; χC_37_-C_36_-C_35_-C_34_ = 8.2°; χC_36_-C_35_-C_34_-C_31_ = 38.1°.

Each of the (*E*,*E*)- and (*E*,*Z*)-isomers being the dication (**1**)^2+^ can exist as a mixture of conformers, resulting from rotation around the single C–C and double C=C bonds between the aromatic rings and rotation of the *N*-ethyl groups. Analysis demonstrates that the arrangement of C=C bonds in [(*E*,*E*)-**1**]^2+^ can be either parallel or V-shaped. In the former case, the symmetry of the conjugated chromophore is *C*_2_; this form will be called the *anti*-[(*E*,*E*)-**1**]^2+^ conformation (Figure 9a). In the latter case, the symmetry is *C*_s_; this conformation will be referred to as *syn-*[(*E*,*E*)-**1**]^2+^ (Figure 9b). In their turn, the ethyl groups at the nitrogen atoms can be either *anti*- or *syn*-oriented relative to the plane of the conjugated chromophore, depending on whether both C-Me groups are above (or below) the plane or one group is above and one group is below the plane. The calculation of all conformers of [(*E*,*E*)-**1**]^2+^ with full geometry optimization demonstrated that the conformers with the *anti*- and *syn*-orientations of the C=C bonds of the conjugated chromophore actually correspond to two local potential energy minima, whereas the C-Me groups can be only *anti*-oriented, since the calculation for the *syn*-C-Me groups revealed a single negative vibration frequency. The same is true for the [(*E*,*Z*)-**1**]^2+^-isomer, which can be formed from *anti*-[(*E*,*E*)-**1**]^2+^ or *syn*-[(*E*,*E*)-**1**]^2+^ via the rotation around the C=C bond by 180°. Therefore, in the subsequent calculations, all C-Me groups were given the *anti*-orientation. The relative stabilities of the *anti*- and *syn*-conformers, as well as the (*E*,*E*)- and (*E*,*Z*)-isomers, of (**1**)^2+^ were calculated.

The bond lengths and dihedral angles in the carbon-nitrogen skeleton of [(*E*,*E*)-**1**]^2+^ and [(*E*,*Z*)-**1**]^2+^ calculated by DFT method with full geometry optimization are shown in Figure 9.

The dication [(*E*,*E*)-**1**]^2+^ is a planar lengthy species, whereas the (*E*,*Z*)-isomer is non-planar because of the steric repulsion between the central benzene ring and the pyridinium ring, which is minimized mainly due to the deviation of the double bond plane (χ_C20-C30-C31-C35_) from the planes of the benzene (χ_C24-C20-C30-C31_) and pyridinium (χ_C30-C31-C34-C36_) rings. Comparison of the data demonstrates that the change in *anti-syn*-conformation has a slight effect on the energy of the molecule.

The energy characteristics are summarized in Table 1.

Comparison of the energies of *anti*- and *syn*-conformers shows that their total energies are equal to the fourth decimal place, with Δ*E* equal to 0.1 kcal/mol being smaller than *kT* at 20 °C (0.582 kcal/mol). Hence, these conformers are equivalent in energy, and at ambient temperature, they are present in solution in approximately equal amounts.

The [(*E*,*Z*)-**1**]^2+^-isomers are energetically less favorable than [(*E*,*E*)-**1**]^2+^ by approximately 5 kcal/mol. The calculation predicts a blue shift of absorption λ_max_ of [(*E*,*Z*)-**1**]^2+^ relative to [(*E*,*E*)-**1**]^2+^, which corresponds to a rather small (0.2 eV) increase in the excitation energy on going from *E* to Z-isomer. A blue shift of λ_max_ corresponds to the experimental data.

We assume that {[(*E*,*E*)-**1**](ClO_4_)_2_} can undergo two-step dissociation in aqueous solutions:

(2) $$(1)(\mathrm{Cl}O_{4}{)}_{2}⇄[(1)\mathrm{Cl}O_{4}{]}^{+}\;+\;\mathrm{Cl}O_{4^{-}}\;⇄(1{)}^{2^{+}}\;+\;2\mathrm{Cl}O_{4^{-}}$$

Although the equilibrium constants are unknown, general considerations suggest that the species predominating in solution is the positively charged ion pair (IP) [(**1**)ClO_4_]^+^ in which the perchlorate ion is located, according to quantum chemical data, over one of pyridine cycles. The IP formation energy, Δ*E*, calculated as the difference between of [(**1**)ClO_4_]^+^ *E*_total_ and the sum of *E*_total_ of (**1**)^2+^ and ClO_4_^–^ is −5.7 kcal/mol (Table 1). In accordance with the small value of Δ*E*, the effect of the counter-ion on the geometrical parameters of chromophors is insignificant. Therefore the IP structures are not shown and were not considered in further calculations.

#### 2.3.2. *E*-*Z* Isomerization in the Excited State

It is generally believed that the *E*-*Z* isomerization occurs through mutual rotation of parts of the molecule around the C=C bond. As this takes place, the corresponding dihedral angle, χ, changes from 180° to 0°. In the ground state, such a rotation is known to be hindered by a high potential barrier. Therefore, the *E*,*Z* isomerization is practically not observed. A different picture occurs in an excited state, in which *E*-*Z* isomerization occurs with varying efficiency. A large number of publications have been devoted to the study of the mechanism of *E*-*Z* isomerization of compounds with a double C=C bond conjugated with aromatic substituents. A clear example is stilbene derivatives, in which isomerization is considered as a photoprocess proceeding via a singlet mechanism [37]. Later, a singlet isomerization mechanism was considered for (**1**)^2+^ in MeCN solution [24]. However, mechanisms of photoprocesses in diazadistyrylbenzenes in aqueous solutions under laser pulse excitation may differ significantly. Indeed, laser excitation leads not only to the formation of the (*E*,*Z*)-**1**-form, but also to the formation of a relatively long-lived photoproduct in the triplet state. An example of such a photoproduct can be a triplet biradical-(**1**)^2+^**,** which is formed through S_1_–T intersystem crossing of excited (**1**)^2+^as a result of electron de-pairing of the C=C double bond. Therefore, calculations of the triplet biradical-(**1**)^2+^ were carried out.

**Figure 10:**
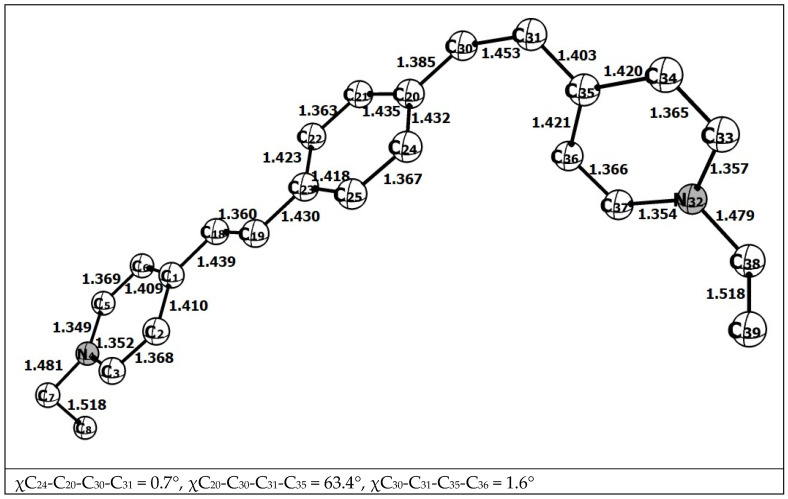
The structure of the triplet biradical calculated with full optimization by DFT/D3 method.

Its energy level was localized to 43 kcal/mol higher than the energy of the ground state (**1**)^2+^ (Table 1). The structure of the biradical calculated by DFT is shown in Figure 10.

**Figure 11:**
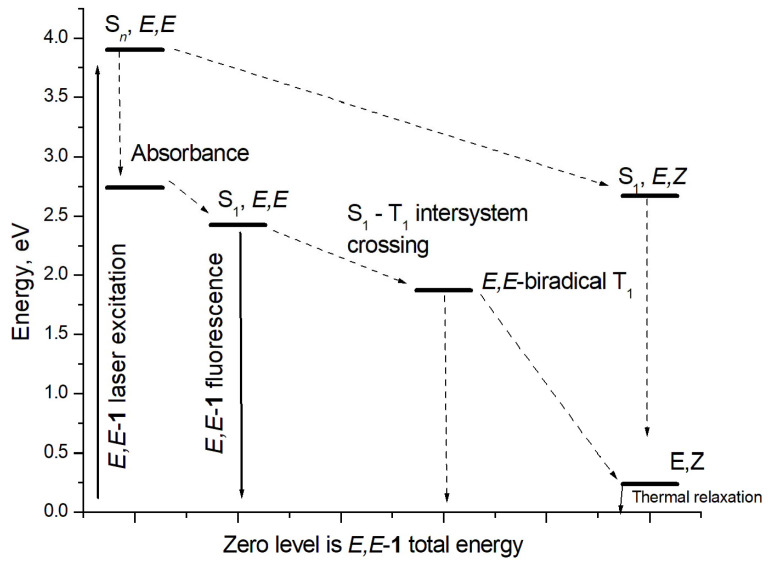
The scheme of photochemical processes of [(*E*,*E*)-**1**]^2+^ calculated by DFT and TDDFT methods.

The maximum of spin population (SP) is localized at C atoms 30 and 31 (0.486 and 0.607). The dihedral angle that ensures repulsion of radical centers with the same spins is 63.4°. This is less than 90°, at which the repulsion appears to be at its maximum and is a consequence of conjugation of the biradical center with the right and left parts of (**1**)^2+^. Among the structural features, the elongated bond C30–C31 1.453 Å should be noted, as it was the C=C double bond in the structure of the ground state [(*E*,*E*)-**1**]^2+^. The scheme of photochemical isomerization is shown in Figure 11.

Two isomerization pathways are shown here. The first, possibly due to the large excess energy of the laser pulse, is the direct isomerization of highly excited [(*E*,*E*)-**1**]^2+^ to [(*E*,*Z*)-**1**]^2+^ in the S_1_ state [24], followed by intramolecular relaxation to the [(*E*,*Z*)-**1**]^2+^ ground state. The second is a lower-energy intersystem transition S_1_–T of the excited state of [(*E*,*E*)-**1**]^2+^ to the triplet one, followed by a return to the singlet ground state. An additional argument in favor of the second pathway is the similarity of the carbon skeleton of triplet biradical (**1**)^2+^ to the cis-form (compare Figure 9c with Figure 10), which should apparently favor the formation of the (*E*,*Z)*-form as a result of the T_1_–S_0_ intersystem transition due to the pairing of electrons in a biradical pair.

#### 2.3.3. Calculation of the Formation of Stacking Dimers of [(*E*,*E*)-**1**]^2+^

The temperature and concentration dependance of the absorption spectra (Figure 5) indicate that [(*E*,*E*)-**1**]^2+^ in the ground state tends to dimerize in aqueous solutions. In our opinion, the major role in the formation of dimers is initially played by hydrophobic interactions, which are manifested as follows. Water, as a solvent, is a three-dimensional network of constantly breaking and forming hydrogen bonds between molecules [38]. The dissolution of [(*E*,*E*)-**1**](ClO_4_)_2_ is accompanied by the formation of hydrates, which represent cavities accommodating each component of equilibrium (2). These cavities are formed upon the breaking of the hydrogen bond network, leading to an increase in the total energy of the system. A decrease in energy can be attained by aggregation, in particular, dimerization of solvates, owing to the fact that the volume of a cavity accommodating a dimer is smaller than the sum of the volumes of two cavities cut out by monomers; that is, dimerization decreases the number of defects in the three-dimensional H-bond network:

(3) $$2\{[E,E-1]\mathrm{Cl}O_{4}{\}}^{+}\;⇄\;\{[E,E-1{]}_{2}(\mathrm{Cl}O_{4}{)}_{2}{\}}^{2^{+}}$$

Quantum chemical DFT calculations of the formation of stacking dimers of [(*E*,*E*)-**1**]^2+^ were carried out with account of implicit consideration of the solvent (water). In the framework of the SMD model [39], which is a part of the DFT procedure (1), we calculated the volume of the cavity occupied by the species and the energy loss by the H-bond network upon the hydrate formation (cavitation energy). According to the calculations with full geometry optimization and at which the *N*-ethyl groups were *syn*-oriented, the cavity volumes for the monomer {[(*E*,*E*)-**1**]ClO_4_}^+^ and dimer **{**[(*E*,*E*)-**1**]_2_(ClO_4_)_2_}^2+^ are 3009.3 and 5954.9 Å^3^, respectively. In other words, the volume of the cavity occupied by the dimer is actually 63.7 Å^3^ smaller than twice the cavity volume occupied by the monomer. This corresponds to an energy decrease upon dimerization by 3.6 kcal/mol. The calculation without SMD procedure demonstrates no dimer formation. Although these values look low, they clearly demonstrate the role of hydrophobic interactions in dimer formation.

Once the dissolved species are brought close together in the bulk solvent, stabilization and structuring of the dimer are due to the electrostatic interaction (EI) and π-stacking of the monomers, resulting from orbital interactions (the term *E*_DFT_ in Formula (5)), and to dispersion forces calculated as Van der Waals (VdW) interaction (term D3 in Formula (5)). When dimerizing ion pairs that are monocations, EI should lead to the repulsion of the components; however, the repulsion is weakened both by the high dielectric constant of water and by the influence of the counterion, ClO_4_^−^. In turn, π-stacking and VdW interactions are favorable for the binding of the monomers. The former type of interaction determines the sandwich **1** orientation, while the VdW interaction, which is isotropic [40], ensures the overall binding in the dimer.

[(*E*,*E*)-**1**]^2+^ is represented by two conformers that are present in the solution in approximately equal amounts. Therefore, during dimerization, five types of dimers can be formed: {*anti*-[(*E*,*E*)-**1**]_2_}^4+^ and {*syn*-[(*E*,*E*)-**1**]_2_}^4+^ with parallel and antiparallel orientation of monomers, as well as a mixed dimer {*anti*,*syn*-[(*E*,*E*)-**1**]_2_}^4+^. In order to minimize BSSE, we calculated five dimer structures while accounting for the gCP correction [41], which turned out to be small (±1.1 kcal/mol) compared to *E_d_* and *E_d_. Therefore, the influence of counterions on *E_d_* and *E_d_ is considered without taking gCP into account. (Table 2).

The calculation showed that, firstly, the parallel dimer of the *syn*-conformer has the best formation energy. Secondly, comparison of the data in Table 1 and Table 2 shows that, in agreement with this experiment, the absorption band of the dimer undergoes a bathochromic shift relative to λ_max_ of monomer [(*E*,*E*)-**1**](ClO_4_)_2_. Comparison of *E*_d_ and *E_d_ with Δ*D* demonstrates that dimers in both the S_0_ and S_1_ states are stabilized by mainly disperse interactions because the absolute values of Δ*D* are higher than *E*_d_ and *E_d_. Upon the S_0_ ⟶ S_1_ transition, both energetic parameters are increased. However, *E_d_ turns out to be greater in magnitude than *E*_d_ by nearly one-third, due to the electronic redistribution, while Δ*D* increases by only 4 kcal/mol.

Thus, the calculation demonstrates the prevailing role of the dispersion interaction for monomer dimerization in an aqueous solution. That is, *EI* in combination with the π-stacking does not ensure binding of the dimer components because of repulsion between the positively charged IPs. The presence of ClO_4_^−^ as a counter-ion stabilizes the dimer by ≈2 kcal/mol. This follows from comparison of *E*_d_ and *E_d_ for {[(*E*,*E*)-**1**]_2_}^4+^ and {[(*E*,*E*)-**1**]_2_(ClO_4_)_2_}^2+^ in the ground and excited states, respectively (Table 2).

**Figure 12:**
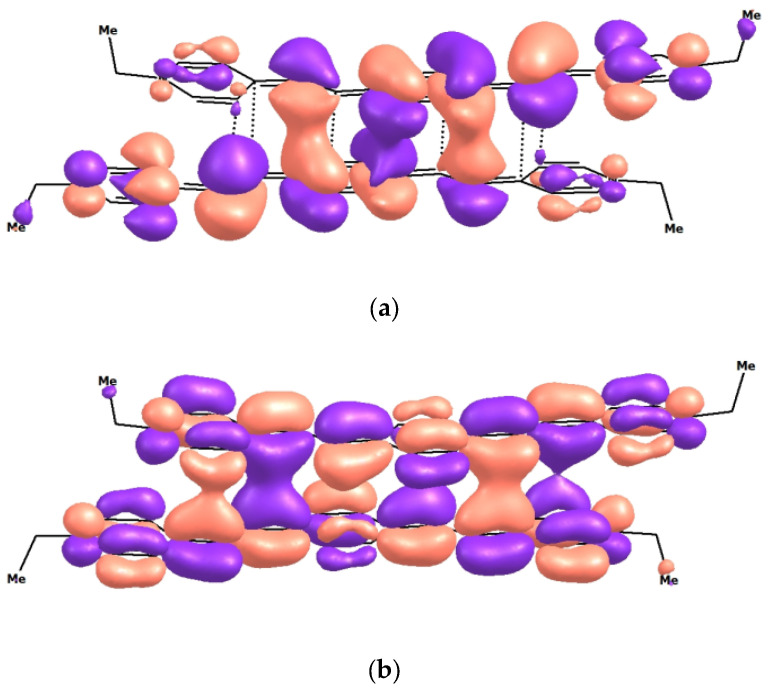
Structure of frontier HOMO of the dimer {[(*E*,*E*)-**1**]_2_}^4+^ in the ground state, S_0_ (**a**), and excited state, S_1_ (**b**), calculated by DFT and TDDFT methods, respectively.

Comparison of the structures of the frontier HOMO of the dimer in the ground (S_0_) and excited (S_1_) states reveals the role of orbital interaction (π-stacking) for the dimer stabilization. It is caused by an increase in the binding character of HOMO between the chromophore planes upon excitation (Figure 12).

**Figure 13:**
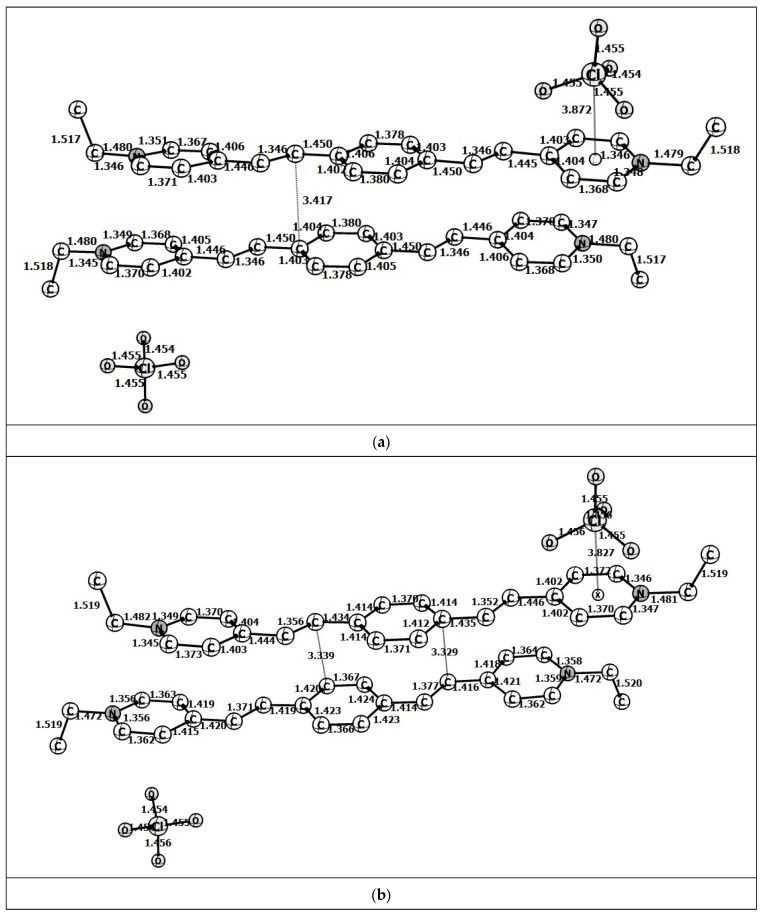
Structures of the {[(*E*,*E*)-**1**]_2_(ClO_4_)_2_}^2+^ dimer in the ground state, S_0_ (**a**), and excited state, S_1_ (**b**), calculated by DFT and TDDFT methods, respectively. The hydrogen atoms are omitted. The distances between the chromophore planes and between [(*E*,*E*)-**1**]^2+^ and ClO_4_^−^ are shown by vertical lines.

Indeed, comparison of the HOMO structures in the ground (S_0_) and excited (S_1_) states indicates that the area of binding (π-stacking) between the monomer planes increases in the S_1_ state. The structures of ground-state and excited-state dimers are shown in Figure 13 (the hydrogen atoms are omitted).

It can be seen from Figure 13 that in the S_0_ structures, the monomers are shifted relative to each other by approximately one bond in such a way that the upper/lower atom of the double bond is projected onto the C atom of the lower/upper central aromatic ring. In the S_1_ structure, the monomers are more shifted relative to each other, in particular, by approximately two bonds, in such a way that the upper/lower double bond is projected onto the C-C bond of the lower/upper central aromatic ring. The overall monomer displacement upon excitation is ~1.4 Å. Simultaneously, the distance between the monomers slightly decreases. Thus, {[(*E*,*E*)-**1**]_2_(ClO_4_)_2_}^2+^ is a VdW dimer that is able to exist only in aqueous solutions. This conclusion is supported by the fact that in MeCN, which has a markedly lower polarity (*ε* = 23) than water, [(*E*,*E*)-**1**](ClO_4_)_2_ does not form dimers [24].

Comparison of the absorption peaks (Table 1) indicates that λ_max_ for {[(*E*,*E*)-**1**]ClO_4_}^+^ is 477 nm, whereas for the {[(*E*,*E*)-**1**]_2_(ClO_4_)_2_}^2+^ dimer, the absorption maximum is at 516 nm. That is, the red shift is 39 nm, which corresponds to a 0.24 eV decrease in the excitation energy. The experimental red shift is 30 nm, or the decrease in the excitation energy is also approximately 0.24 eV. In addition, the calculation shows an increase in the dimerization energy (in magnitude) upon excitation; this is consistent with the laser photolysis data, which demonstrate a photoinduced increase in the absorbance in the dimer absorption region. Hence, the calculation results are in good agreement with experimental data that provided the conclusion about the presence of [(*E*,*E*)-**1**]_2_(ClO_4_)_2_ dimers in solution.

#### 2.3.4. Inclusion Complexes of the Dye [(*E*,*E*)-**1**]^2+^ with CB[7]

**Figure 14:**
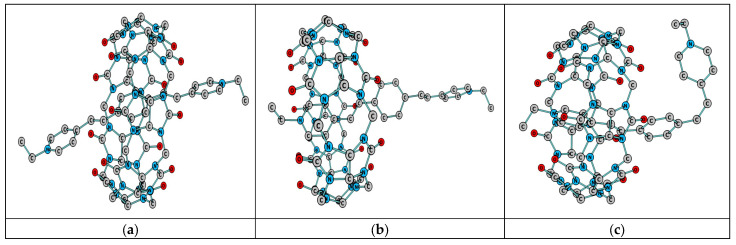
Structures calculated by DFT-D3 method: (**a**) {[(*E*,*E*)-**1**]@CB[7]_c_}^2+^, S_0_; (**b**) {[(*E*,*E*)-**1**]@CB[7]_r_}^2+^, S_1_; (**c**) {[(*E*,*Z*)-**1**]@CB[7]*_e_*}^2+^, S_1_.

Cucurbit[7]uril is a macrocyclic compound that is known to form inclusion complexes with organic molecules in water solutions. An increase in the fluorescence intensity of **1** in the presence of CB7 is evidence of the formation of a complex (Figure 4b). The length of the [(*E*,*E*)-**1**]^2+^ dication, considering the VdW radii of the constituent atoms, is ~25 Å, and the distance between the oxygen atoms of CB[7] portals is 9 Å. Therefore, during the formation of the inclusion complex, the CB[7] may occupy a position either in the middle of {[(*E*,*E*)-**1**]@CB[7]_c_}^2+^ (Figure 14a) or at the edge, {[(*E*,*E*)-**1**]@CB[7]_e_}^2+^ (Figure 14b). A calculation shows that the former is energetically more favorable than the latter by 5.6 kcal/mol in the ground state. But in the excited state, complex {[(*E*,*E*)-**1**]@CB[7]_e_}^2+^ turns out to be more favorable by 9.6 kcal/mol than {[(*E*,*E*)-**1**]@CB[7]_c_}^2+^. Comparison of *E*_c_ and *E_c_ with ΔD shows that both the *E*_c_/ΔD and *E_c_/ΔD ratios are in the range of 2.5 ÷ 3.1; thus, the *E*_c_ and *E_c_ values consisting of the electrostatic interactions plus ΔD are much greater than the pure dispersion contribution. It follows that the inclusion complexes of **1**@CB[7] can be considered as donor–acceptor compounds in which the CB[7] is an electron-rich molecule due to the presence of two portals consisting of sixteen C=O groups carrying a partial negative charge. Meanwhile, [(*E*,*E*)-**1**]^2+^, being a dication, is electron-deficient.

The calculation also shows (Table 3) that, upon the inclusion of the [(*E*,*E*)-**1**]^2+^ dication into the CB[7] cavity to give the complex {[(*E*,*E*)-**1**]@CB[7]_c_}^2+^, the positive charge of the [(*E*,*E*)-**1**]^2+^ dication is reduced from +2 to +1.9 ē, meaning that the charge transferred from CB[7] to [(*E*,*E*)-**1**]^2+^ is 0.1 ē, neutralizing dication partially. For the {[(*E*,*Z*)-**1**]@CB[7]*_e_*}^2+^ complex, the charge transfer is larger and amounts to 0.185 e. In excited complexes, charge transfer tends to increase, contributing to an increase in the stability of the complexes.

#### 2.3.5. Photoinduced Displacement of (**1**)^2+^ in the Cavity upon Transition to the Excited State

The photoinduced migration of the [(*E*,*E*)-**1**]^2+^ dication inside the CB[7] cavity is of interest for the design of molecular machines. Since [(*E*,*E*)-**1**]^2+^ is a lengthy species, it might be expected that upon its excitation and accompanying change in the electron density distribution, the relative arrangement of the components of the complex may change, while upon returning to the ground state, the initial structure of the complex would be restored.

The main result of laser photolysis is *E-Z* isomerization, in which the elongated [(*E*,*E*)-**1**]^2+^ dication is converted into the bent [(*E*,*Z*)-**1**]^2+^ one. When the same reaction occurs inside the CB[7] cavity for the {[(*E*,*E*)-**1**]@CB[7]*_c_*}^2+^ complex, its components must change their relative positions due to the limited size of the cavity, which does not accommodate the bent structure. According to calculations, the most likely mechanism for such a transformation could be as follows. When transitioning to an excited state, the dication tends to partially exit the cavity, shifting by 5.2 Å, after which the protruding fragment undergoes *E-Z* isomerization, i.e., a sequence of reactions occurs (Figure 14):

14a ⟶ 14b ⟶ 14c ⟶14a(4)

This assumption is based on the fact (Table 3) that in an excited state, [(*E*,*E*)-**1**]^2+^ with a partially exited dication, {[(*E*,*E*)-**1**]@CB[7]*_e_*}^2+^ is energetically more favorable than [(*E*,*E*)-**1**]^2+^ with a central location, {[(*E*,*E*)-**1**]@CB[7]*_c_*}^2+^. In turn, the bent (*E*-*Z*)-isomer in the cavity, {[(*E*,*Z*)-**1**]@CB[7]}^2+^, is energetically more favorable than {[(*E*,*E*)-**1**]@CB[7]*_e_*}^2+^. Upon returning to the ground state, the (*E*-*Z*)**-**isomer undergoes a transformation into the initial *E*-*E* isomer due to slow thermal relaxation resulting in {[(*E*,*E*)-**1**]@CB[7]*_c_*}^2+^.

## 3. Materials and Methods

*The synthesis* of [(*E*,*E*)-**1**](ClO_4_)_2_ is beyond the scope of this study and will be published elsewhere. ^1^H-NMR and ^13^C-NMR spectra of [(*E*,*E*)-**1**](ClO_4_)_2_ can be found in Supplementary Materials Figures S1 and S2. The absorption spectra of dye [(*E*,*E*)-**1**](ClO_4_)_2_ in the ground state were recorded on an Agilent 8453 spectrophotometer (Agilent Technologies, Santa Clara, CA, USA) in standard (1-cm thick) quartz cells. Luminescence spectra were recorded on a Varian Eclipse spectrofluorometer, (Agilent Technologies, USA). Dissolved oxygen was removed by bubbling argon through the solution. Cucurbit[7]uril CB[7] (Aldrich, St. Louis, MO, USA) was used as the cavitand. All measurements were carried out at room temperature. Deionized water (Millipoure, Burlington, MA, USA) served as a solvent. The absorption spectra and transformation kinetics of photoreaction intermediates were measured on a pulsed-laser photolysis setup. A neodymium laser on an yttrium aluminum garnet (Nd-YAG) (Solar Laser Systems, Minsk, Belarus, LQ529A; λ = 355 nm, energy per pulse of 70 mJ) was used as the laser radiation source. The pulse duration was 10 ns. The system for spectral detection of photoreaction intermediates included an Osram-150 xenon lamp with an electric power unit (Applied Photophysics, Leatherhead, UK) as a probe light source, an MDR-6 double monochromator, a FEU-84 photomultiplier, a digital oscilloscope (Bordo-211 board), and a personal computer.

*Quantum chemical calculations* were carried out using an ORCA Version 6.0.1 program package [42] by DFT and TDDFT methods with the B3LYP functional including the D3(BJ) dispersion corrections (below referred to as D3), the TZVP triple-zeta basis set, and implicit consideration of the solvent (water) using the SMD model [39] embedded in the DFT calculation procedure. It is noteworthy that the calculation of dimers without taking into account the implicit influence of the solvent does not show their formation. Moreover, in order to minimize BSSE at the calculation of dimers and inclusion complexes, we used procedure gCP [41]. Thus, the total energy was calculated by Formula (5) [40]:

*E*_total_ = *E*_DFT_ + *E*_disp_ (D3) + gCP (5)

To test the ability of the B3LYP method to correctly predict the energy difference between the (*E*,*Z*)-isomers at the C=C double bond, we calculated the classical (*E*,*Z*)-isomer pair of butene-2. B3LYP calculations using the TZVP basis set showed that the *E* isomer is 1.29 kcal/mol more energetically favorable than the *Z* isomer. Since the experimental value is 1.13 kcal/mol [43], our calculation method is adequate for the problem, and we used it without comments.

The dimerization energy for the stacking dimers in the S_0_ state (*E*_d_) was calculated as the difference between the optimized total energy of the dimer and twice the optimized total energy of the monomer, *E*_m_. The dimerization energy for the stacking dimers in the S_1_ state (*E_d_) was calculated by Formula (6):

*E_d_ = *E_total_(dimer) − *E*_m_ − *E_m_ (6)

where *E_total_(dimer) is the optimized total energy of the dimer, and *E*_m_ and *E_m_ is the optimized total energies of the monomers in the ground and excited states, respectively.

The use of time-dependent DFT for calculating the vertical excitation energy with the B3LYP functional is justified by the fact that, according to the benchmarks [44], an average error is of 0.13 eV.

## 4. Conclusions

The dimerization and *E*-*Z* isomerization reactions of the 1,4-diazastyryl)benzene derivative {[(*E*,*E*)-**1**](ClO_4_)_2_} were established on the basis of experimental data on absorption and fluorescence spectroscopy, as well as pulsed-laser photolysis. The dimerization [(*E*,*E*)-**1**](ClO_4_)_2_ in water solution is due to the interplay of two factors. The hydrophobic interaction acts as an external factor that promotes the approach of monomers in solution, while quantum chemical calculations show that π-stacking and dispersion interaction act as internal factors, providing the necessary mutual orientation and binding of components, and that the dimerization of the dye is energetically more favorable in the excited state S_1_. Calculations also show that there are two possible pathways for *E-Z* isomerization via the singlet and triplet states of [(*E*,*E*)-**1**](ClO_4_)_2_. In aqueous solution, [(*E*,*E*)-**1**]^2+^ forms a 1:1 donor–acceptor inclusion complex with the cavitand cucur[bit-7]uril (CB[7]), in which CB[7] is the donor, and the dye dication is the acceptor. Reversible photoinduced migration of the dye [(*E*,*E*)-**1**]^2+^ dication in the CB[7] cavity occurs as a result of partial exit of the [(*E*,*E*)-**1**]^2+^ from the cavity and its subsequent conversion to the *E*,*Z* isomer in the excited state. After returning to the ground state, the (*E*,*Z)*-isomer undergoes conversion to the initial complex with CB[7] centrally located relative to the [(*E*,*E*)-**1**]^2+^. This process is of interest in relation to the scientific task of designing photocontrolled supramolecular machines.

## Figures and Tables

**Table 1:** Total energies of monomers, ion pairs ([(**1**)ClO_4_]^+^,.IP), and biradical (*E*_total_), relative energies of the isomers and conformers of (**1**)^2+^, (Δ*E*), IP formation energy. The λ_max_ and *E*_excit_ values calculated by TDDFT.

Monomers, Conformers, Ion Pairs, Biradical	*E*_total_, a.u.	Δ*E*, kcal/mol	λ_max_, nm	*E*_excit_, eV
*syn*-[(*E*,*E*)-**1**]^2+^	−1039.24591	0 *^a^*	482	2.57
*anti*-[(*E*,*E*)-**1**]^2+^	−1039.24581	0.1*^a^*	478	2.59
biradical-(**1**)^2+^ T_1_	−1039.17707	43.1 *^b^*		
*syn*-[(*E*,*Z*)-**1**]^2+^	−1039.23720	5.5*^a^*	450	2.76
*anti*-[(*E*,*Z*)-**1**]^2+^	−1039.23719	5.4*^a^*	451	2.75
{[*syn*-(*E*,*E*)-**1**]ClO_4_}^+^ {[(*syn*-*E*,*Z*)-**1**]ClO_4_}^+^	−1800.23224 −1800.22394	−5.7 *^c^* −5.2 *^c^*	477	2.60

*^a^* Energy difference between *syn*–*anti* conformers for (*E*-*E)* and (*E*-*Z)* isomers.*^b^* Energy level of the triplet biradical relative to the energy of the *syn*-[(*E*,*E*)-**1**]^2+^ in ground state.*^c^* IP formation energy, kcal/mol.

**Table 2:** Total energies of dimers, (*E*_total_); dimerization energies (*E_d_* and *E_d_); contribution of the dispersion corrections to *E*_d_ and *E_d_ (Δ*D*); λ_max_; and excitation energy.

Dimers	*E*_total_, a.u.	*E_d_* and *E_d_, kcal/mol	Δ*D* kcal/mol	λ_max_, nm	*E*_excit_, eV
*Paral*-(*syn*,*syn*{[(*E*,*E*)-**1**]_2_}^4+^), S_0_	−2078.53239	−25.4	−36.2		
*Paral*-(*syn*,*syn*{[(*E*,*E*)-**1**]_2_}^4+^), S_1_	−2078.45831	−34.2	−40.2	493	2.52
*Paral*-(*syn*,*syn*{[(*E*,*E*)-**1**]_2_(ClO_4_)_2_}^2+^), S_0_	−3600.50849	−27.6	−36.5		
*Paral*-(*syn*,*syn*{[(*E*,*E*)-**1**]_2_(ClO_4_)_2_}^2+^), S_1_	−3600.43428	−36.5	−40.9	516	2.40

**Table 3:** Total energies of inclusion complexes (*E*_total_), complexation energies of (**1**)^2+^@CB[7] in the ground and excited states (*E*_c_ and *E_c_), and contribution of the dispersion corrections (Δ*D*) to *E*_c_ and *E_c_. Charge of (**1**)^2+^ in the complex (Q((**1**)^2+^)) and charge transfer from CB[7] to [(*E*,*E*)-**1**]^2+^, Δq. λ_max_ and excitation energy.

Inclusion Complexes	*E*_total_, a.u.	*E*_c_ and *E_c_, kcal/mol	Δ*D*, kcal/mol	Q((1)^2+^)/Δq, ē	λ_max_, nm	*E*_excit_, eV
{[(*E*,*E*)-**1**]@CB[7]*_c_*}^2+^*^a^*	−5251.03595	−93.6	−37.1	1.9/0.1		
{[(*E*,*E*)-**1**]@CB[7]*_c_*}^2+^, S_1_	−5250.93640	−96.7	−38.5	1.846/0.154	443	1.06
{[(*E*,*E*)-**1**]@CB[7]*_e_*}^2+^*^a^*	−5251.02700	−88.0	−32.5	1.867/0.133		
{[(*E*,*E*)-**1**]@CB[7]*_e_*}^2+^, S_1_	−5250.94154	−106.3	−34.7	1.818/0.182	466	2.18
{[(*E*,*Z*)-**1**]@CB[7]}^2+^*^a^*	−5251.03285	−99.6	−42.4	1.815/0.185		
{[(*E*,*Z*)-**1**]@CB[7]}^2+^, S_1_	−5250.94820	−110.5	−40.7	1.796/0.204	474	1.28

*^a^ c* and *e* are designations of complexes in which (**1**)^2+^ is inserted into CB[7] by the central and lateral parts, respectively.

## Data Availability

Data are contained within the article and Supplementary Materials.

## Supplementary Materials

The following supporting information can be downloaded at https://www.mdpi.com/article/10.3390/molecules31091464/s1, Figure S1: ^1^H NMR spectrum of [(*E*,*E*)-**1**](ClO_4_)_2_; Figure S2: ^13^С NMR spectrum of [(*E*,*E*)-**1**](ClO_4_)_2_.
