# Supplementary material for: Prospects for the Creation of a Photocontrolled Supramolecular Machine Based on a 1,4-Di(azastyryl)benzene Derivative and Cucurbit[7]uril
Source: Molecules. 2026 Apr 28;31(9):1464. doi: 10.3390/molecules31091464 (PMC13165326; doi:10.3390/molecules31091464)
Supplement: Supplementary file 1 [file molecules-31-01464-s001.zip › molecules-4264295-supplementary.pdf]

NMR spectra of compounds [(*E*,*E*)-1](ClO_4)$_2$

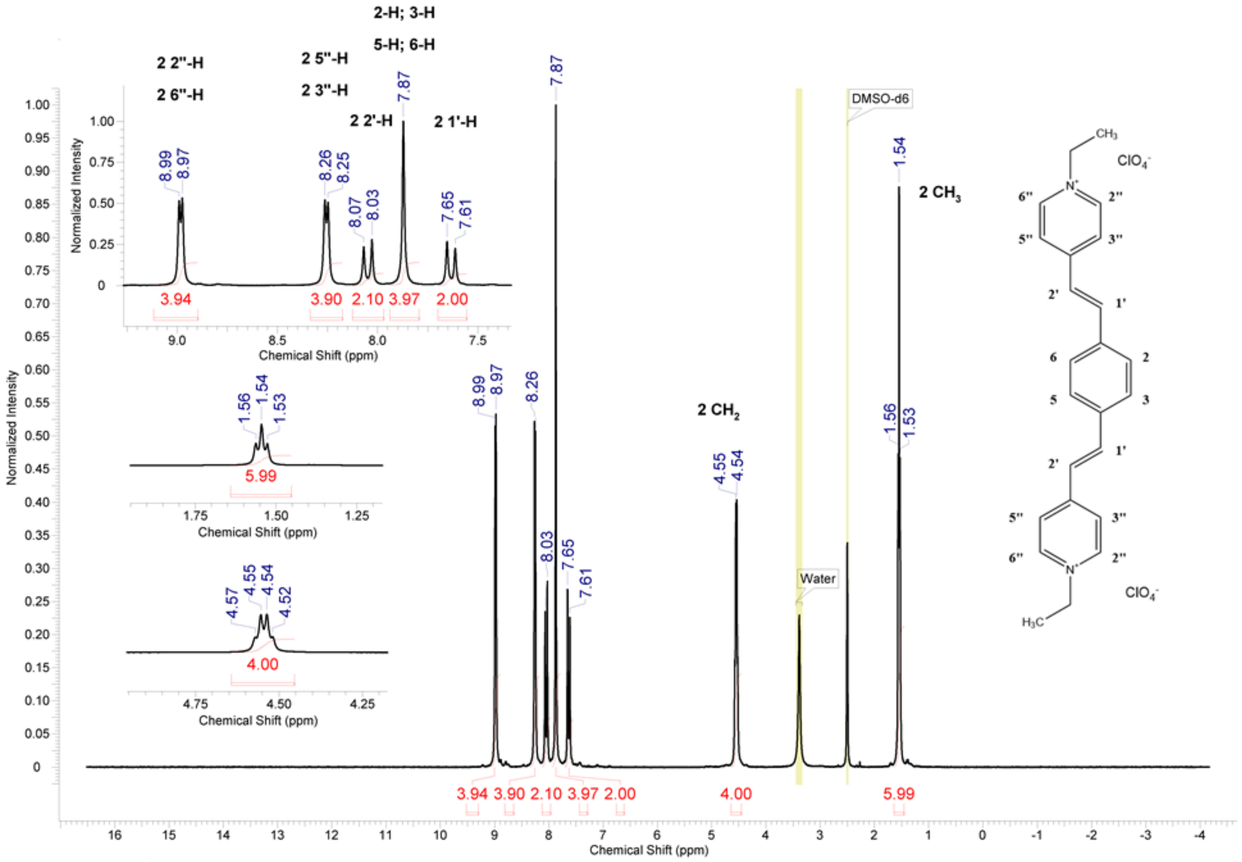

Figure S1. ^{1}H NMR spectrum of [(*E*,*E*)-**1**](ClO_4)$_2$.

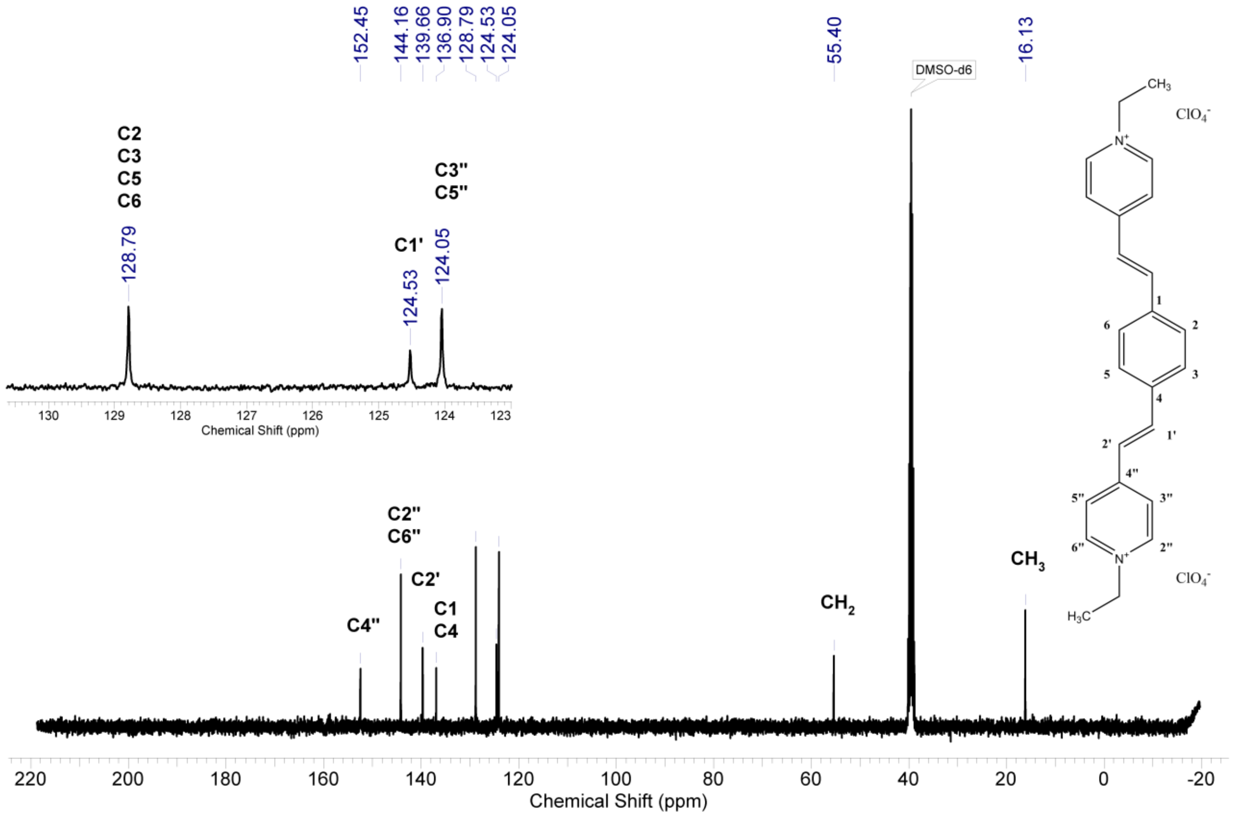

Figure S2. ^{13}C NMR spectrum of [(*E*,*E*)-**1**](ClO_4)$_2$.
